# Clinical experience in the use of 3D printing as a rapid replacement of traditional radiation therapy immobilization materials

**DOI:** 10.1002/acm2.14008

**Published:** 2023-05-02

**Authors:** Eric D. Ehler

**Affiliations:** ^1^ Department of Radiation Oncology University of Minnesota Minneapolis Minnesota USA

**Keywords:** 3D printing, IGRT, immobilization, radiotherapy

## Abstract

**Purpose:**

Patient positioning and immobilization devices are commonly employed in radiation therapy. Unfortunately, cases can arise where the devices need to be reconstructed or improved. This work describes clinical processes to use a planning CT, to design and 3D print immobilization devices for reproducible patient positioning within a clinically feasible time frame when traditional methods can no longer be used or are insufficient.

**Materials/Methods:**

Three clinical cases required rapid 3D printing of an immobilization device mid‐treatment due to the following: (1) a lost headrest cushion, (2) needed improvement in lumbar spine positioning, and (3) a partially deflated vacuum immobilization mattress.

**Results:**

In the three cases, the 3D printed immobilization devices were clinically implemented successfully; two of the devices were fully designed and printed in 1 day. The 3D printed immobilization devices achieved a positioning accuracy sufficient to avoid the necessity to repeat the simulation and planning process.

**Conclusion:**

If traditional immobilization devices fail or are misplaced, it is feasible to have a 3D printed replacement within the time span of 1 day. The design and fabrication methods, as well as the experiences gained, are described in detail to assist clinicians to implement 3D printing for similar situations.

## 1 INTRODUCTION

There is increasing research into the use of 3D printing in radiation oncology.^1^ Specifically, the use of 3D printing in patient immobilization is an area of interest.^2^ Most of the work has focused on immobilization for head and neck treatments either as a head rest,^3^ mask,^4^, ^5^ or oral stent.^6^ Analysis of the accuracy of 3D printed head immobilization has been reported on healthy volunteers.^4^, ^7^ One abstract has compared the accuracy of 3D printed immobilization with standard techniques in actual patient treatments (*N* = 30) with a slight improvement in position accuracy with the 3D printed headrest.^8^ However, most of the previous works^3^, ^4^, ^5^, ^7^, ^9^, ^10^ have not been implemented into clinical practice where tight time constraints are a common factor. Aspects of 3D printing in realistic clinical timeframes and conditions have been previously reported for radiotherapy bolus.^11^

In this work, three instances where standard immobilization was replaced with 3D printed devices are presented. In these cases, rather than performing repeat clinical workup (treatment simulation, contouring, and replanning), 3D printing was utilized to achieve the desired positioning accuracy and immobilization to allow treatment to continue without interruption of the radiation treatment schedule. This work describes the use of 3D printing to produce clinically viable radiotherapy immobilization devices in very short time frames; data on the treatment positioning accuracy during the use of 3D printed immobilization devices are also provided. The cases described here are beneficial not just to clinicians with similar situations but also to those just starting, or considering starting, implementing 3D printing into their clinical workflow.

## 2 METHODS AND MATERIALS

This section describes the steps taken at the time of the procedure and is not necessarily recommended methodology. Detailed descriptions of the methods are provided along with modifications gained in hindsight. The University of Minnesota IRB has determined that this study is not research involving human subjects as defined by DHHS and FDA regulations (IRB ID: STUDY00007327). Two of the cases occurred within a 3‐month period, roughly 3.5 years after the first case. 3D printing statistics such as print time and amount of material were recorded for all of the cases. The time required to generate the 3D models was consistent with previously published^11^ times to generate radiotherapy bolus. All cases generated the 3D model based on CT data with a slice thickness of 3 mm and an in plane voxel size of about 1 mm × 1 mm.

### 2.1 Missing head rest

**FIGURE 1:**
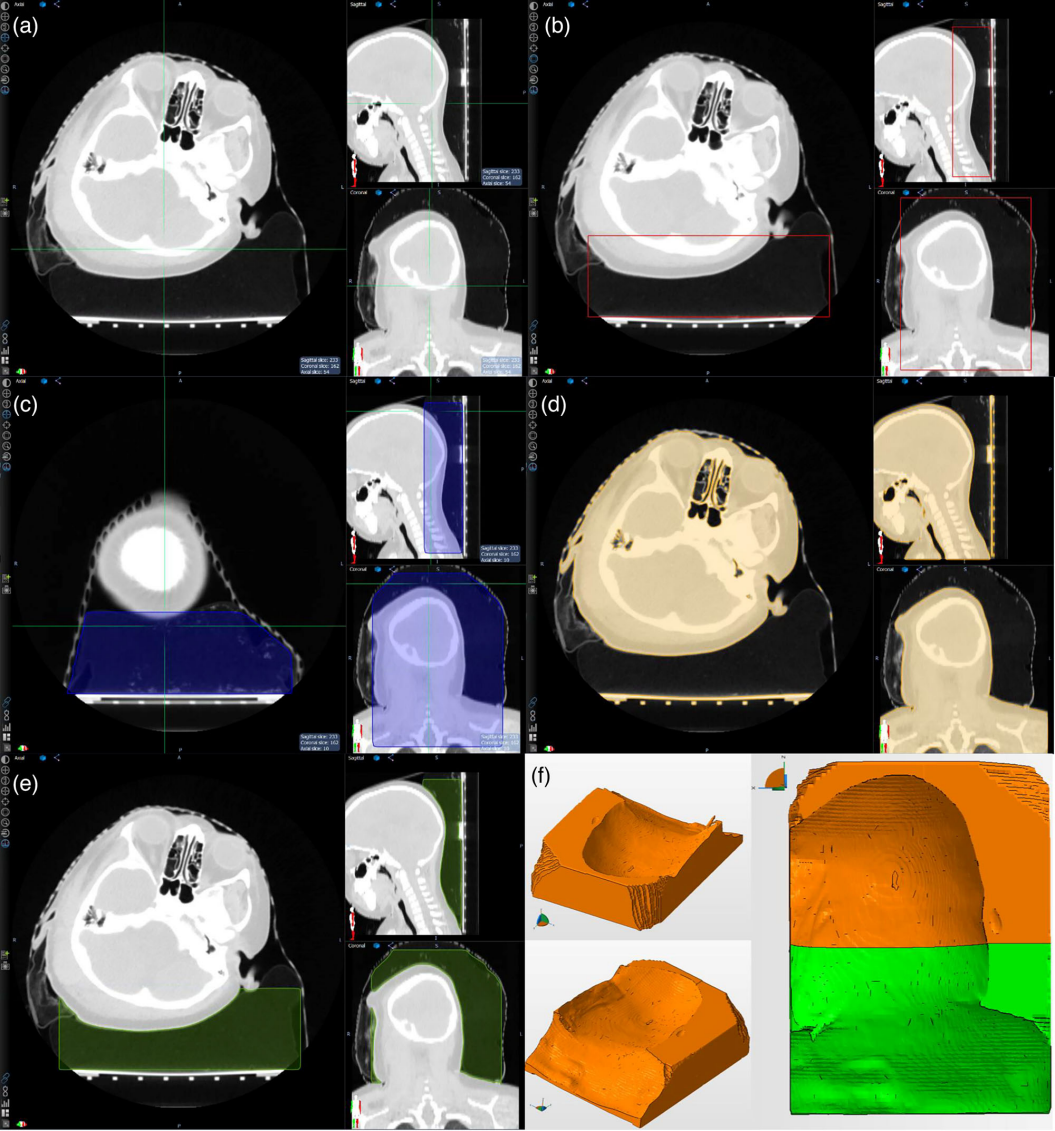
Generation of 3D model for head rest used in a brain treatment shown as follows: (a) planning CT with initial head rest visible, (b) cubic structure that encompasses roughly the region of the head, neck, and shoulders as the original head rest, (c) manual modification of the cubic structure to avoid overlap with the thermoplastic mask, (d) external body image, (e) new structure resulting from subtracting the external contour from the modified cubic structure, and (f) final 3D model is shown in three views, on the right orange (“Part 1″) and green (“Part 2″) regions indicate how the headrest was printed in two parts.

This case was a 3D conformal brain treatment. For immobilization, a thermoplastic mask was used along with a cushioned headrest that was initially soft and formable at the simulation and which hardened over the time span of a few minutes. The need to 3D print a replacement headrest arose when the original headrest was mistakenly discarded with two fractions remaining; the thermoplastic mask was preserved, however. It can be seen in Figure 1a that the planned patient head position is not in a neutral position but skewed looking to the patient's left due to wound healing on the patient's right side. Therefore, reforming a new headrest using the standard technique was determined not likely to be reproducible, and replanning of the remaining two fractions might be required. It was then decided to attempt to recreate the original head rest with 3D printing in order to avoid delaying treatment.

The original planning CT was used as the basis for the 3D model generation. First a cubic structure was created that would encase an equivalent portion of the head and neck as the original head rest (cf. Figure 1b). The height of the headrest was chosen so that the left ear would have slight contact in order to replicate the head tilt. The cubic structure was manually modified in regions where the cube structure would have intersected with the mask in the CT image (cf. Figure 1). Then an external body contour was automatically generated based on the Hounsfield Unit threshold. In this case, an HU of −200 was used as the minimum HU threshold. A threshold of −600 to −400 HU would have been preferable but unintended parts of the original headrest would have been included in the threshold range. It should be noted that an indexing bar and pins were included in the external contour which can be seen posterior to the occipital protuberance in the sagittal view in Figure 1a‐e. The intersecting regions of the cubic structure and external contour were cropped from the modified cubic structure, see Figure 1e. The structure was converted to .STL format. The 3D model was analyzed in 3D modeling software (NetFabb, Autodesk, Inc., San Rafael, CA). Any deficiencies in the model were corrected with an automated 3D model repair module in the software. Finally, the 3D model of the headrest was split into two parts due to the limitations in the maximum print size of the 3D printer available at the time. Hereafter for this case “Part 1” will refer to the orange‐colored region of the right side of Figure 1f and “Part 2” will refer to the green.

The processed 3D model was imported into a slicing program (Makerware, Makerbot LLC., New York, NY) and printed on a Makerbot Replicator 2X. The nozzle size of the printer was 0.4 mm. The vendor recommended polylactic acid (PLA) plastic be used. A layer height of 0.3 mm was used and the infill was 10% with a honeycomb infill pattern. A low infill percentage was used to limit the printing time.

After the headrest was printed, a dry run was performed to ensure proper clearance of the mask and headrest. It was noted at this time that neither the indexing bar nor the pin holes fit; there was not enough clearance in the 3D printed headrest and the devices. The pin holes were successfully reamed out with a drill and provided enough stability and positional accuracy with the mask frame that the indexing bar was deemed unnecessary. It should be noted that none of the treatment beams entered the patient through the 3D printed headrest so the radiation effects of the headrest were not investigated. However, if the entrance beams intersected the 3D printed headrest, the low infill percentage would have minimized the dosimetric impact.^12^, ^13^

Although not initially performed, image guidance with cone beam CT (CBCT) was performed to ensure proper patient alignment on the two fractions using the 3D printed head rest. Image guidance shifts for the two boost fractions are reported in the Section 3. No intracranial anatomy was visible in the CBCT images so the skull was used as a surrogate; the mean surface point distance was calculated for the planning CT and the daily CBCT images using a radiation oncology contouring software environment (Velocity 3.1, Varian Medical Systems, Palo Alto, CA, USA) for the skull contoured in the planning CT and in the two CBCT images.

### 2.2 Lumbar spine support for improved alignment

This case was a helical tomotherapy treatment of the entire spine (36 Gy prescribed dose). Standard immobilization included a thermoplastic head, neck, and shoulder mask. The arms were positioned akimbo and a complete block of the arms was used in the treatment planning software (TPS). Alignment marks were drawn on the mask and at points on the torso around the level of the xyphoid process and inferior of the umbilicus. A vacuum mattress was placed under the legs; it was not used in the pelvis/lower torso region to not interfere with the patient positioning using the in‐room laser projections on the skin markers. Typically, acceptable interfraction reproducibility is achieved with this method and is preferred over a full‐body vacuum mattress in our clinic. During megavoltage CT (MVCT) imaging of the first fraction (about 2 weeks after the CT simulation), it was observed that the arch of the lumbar spine region did not match the position in the planning CT. The patient was repositioned and an improvised cushion was constructed to better support the lumbar spine made of folded towels. The subsequent image showed clinically acceptable alignment and the first treatment fraction was delivered. The daily image registration was tracked for the first six treatments (out of 18 total) and the difficulty in positioning the lumbar region remained despite the clinicians carefully using the same technique to cushion the lumbar spine region (i.e., the same type of towel folded in the same manner). At this point, it was determined that either a technique to improve the time and ease of achieving proper positioning of the lumbar spine arch was needed (often multiple MVCTs were needed for repositioning) or replanning based on a CT with a spine alignment more representative of the daily imaging scans was required. It was determined to try 3D printing lumbar spine support before the next fraction; if the daily imaging was not improved or if the time and effort to achieve an acceptable spine position were not decreased, then the treatment would be paused for replanning. The daily alignment was monitored after the initial use of the 3D printed support to verify acceptable alignment.

**FIGURE 2:**
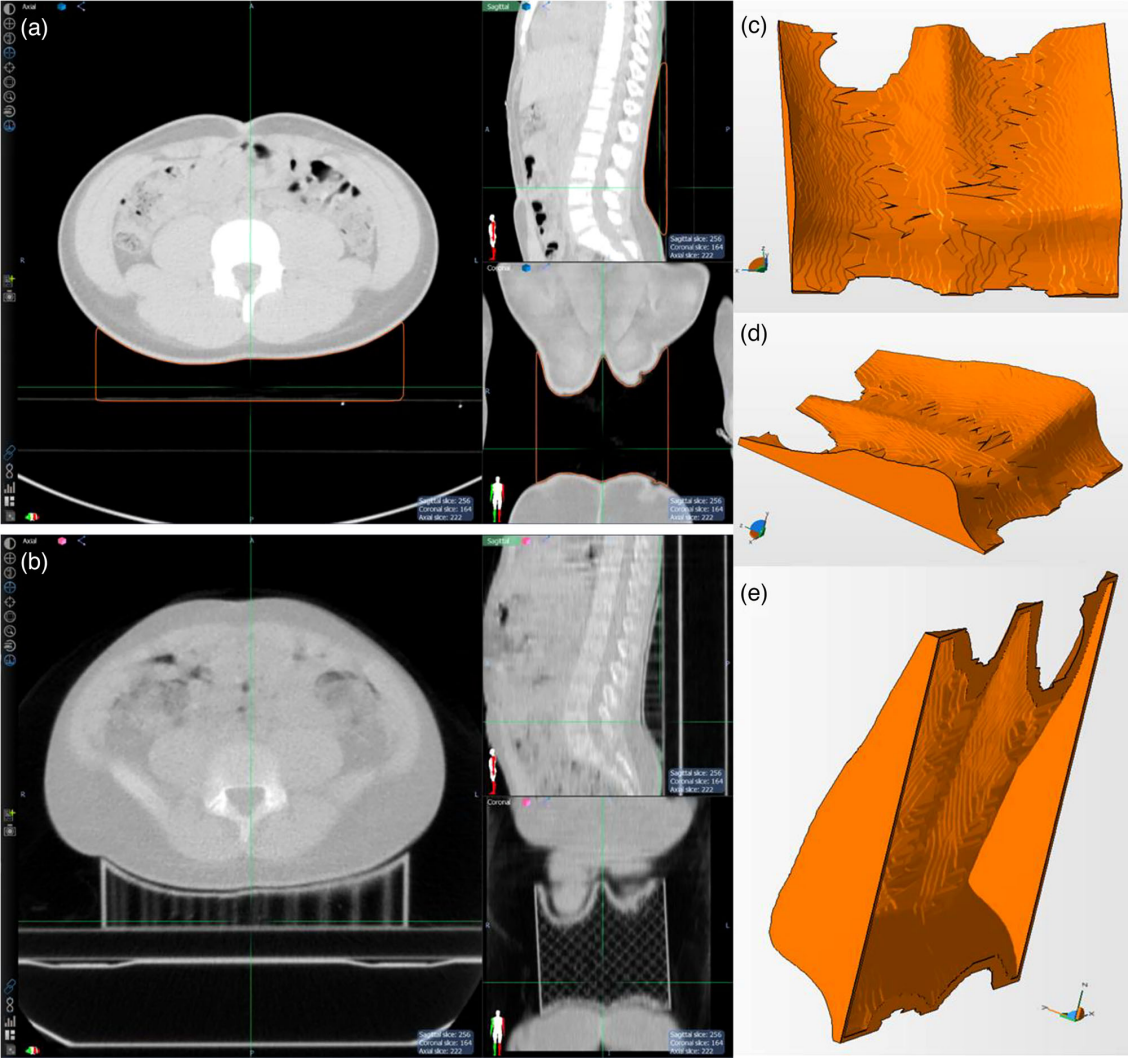
Generation of 3D model for lumbar spine as follows: (a) axial, sagittal, and coronal view of the cubic structure subtracted from the 1 mm external body contour, (b) axial, sagittal, and coronal view of the daily MVCT with the 3D printed lumbar support in place with grid shape support structure, (c) anterior view of the 3D Model, (d) oblique view of the model highlighting the unique shape of the lumbar support, and (e) posterior (underside) of the hollow 3D model (note the support structure was added later by the slicing software).

Similar to the procedure described in Section 2.1, a cubic structure was constructed to provide lumbar support. In this case the cubic structure was set just to the inside of the couch top by about 3 mm. An external contour was created by setting a minimum HU threshold of −400. Unlike the previous case, the external contour was expanded 1 mm prior to cropping it from the cubic structure. Prior to converting the cropped structure to .STL, a smoothing algorithm within Netfabb was applied to eliminate sharp edges that could cause patient discomfort and possible intrafraction movement; the treatment time, including imaging of the entire spine, was around 15–20 min. The 3D model was converted to a 2.4 mm thick hollow shell using a feature within Netfabb. The shell wall that was extended below the couch surface in the planning CT image was cropped from the 3D model to reduce the amount of x‐ray attenuation in the plastic creating an open‐faced shell (cf. Figure 2e); it should be noted the reason the cubic structure was originally set just inside of the couch top was to accommodate the cropping of 3 mm of the posterior model; a grid scaffold, or support, structure with 8% infill was used and created by the slicing software; it was left in place for treatments to provide structural support. At this time a new 3D printer had been obtained with a larger build volume (28 cm × 28 cm × 25 cm). This resulted in the capability of printing the entire lumbar arch support in one piece. The printer was also fitted with a larger nozzle (1.2 mm) than the previous case, allowing for larger layer thicknesses. Polyethylene terephthalate glycol (PET‐G) was used, rather than the PLA used in the first case; experience gained since the first case had shown PET‐G to be an easier material to print with and produced structurally stronger printed parts. The total fabrication turnaround time was 1 day and the device was able to be used the next day (fraction 7) which is less than the time that would have been needed to replan the treatment with a new CT simulation scan.

Daily monitoring of the spine localization was initiated on the first fraction using the planning CT and daily MVCT registration images. First, the spinal canal (which will hereafter be called CTV) was contoured on the planning CT, and all subsequent MVCTs, and then split into segments along each vertebral body (C1 through L5). Next, the daily clinical rigid registrations, using translational and a roll correction only, were applied and the CTVs for the planning image and rigid fusion daily MVCT were compared. Dice coefficients and mean surface point distances were determined for each segment of the CTV associated with its vertebral body. In addition, the whole CTV was expanded with 1, 3, 5, 7, 9, and 11 mm margins. The CTV in the daily MVCT images was Boolean subtracted from the original CTV in the planning CT. The volumes of the daily CTV position outside of the expansions were recorded to give a comparison of the interfraction positioning uncertainty margin. This was performed to ensure that the clinical CTV to PTV margin (1 cm) was adequate and to serve as a reference for future clinical cases.

The dosimetric impact of margin expansions on the CTV was approximated (dose calculation of the non‐clinical margin expansion plans on the daily images is not possible in the TPS). Test plans were created for each expansion distance listed above. The rigid daily MVCT fusion volumes for the spinal canal were evaluated for each of the margin expansions. The planning doses for each margin expansion were propogated onto the daily MVCT using the rigid daily MVCT fusion. The doses were then propogated back to the planning CT using deformable registration to create an approximate composite dose on the planning CT. Attenuation through the couch is accounted for in all plans via a TPS built in module.

After the treatment course was complete, statistical analysis of the Dice coefficient, the mean distance, and the volume receiving >95% of the prescription dose (V95%) was performed using a two tailed, unequal variance *t*‐test.

### 2.3 Deflated vacuum mattress

This case was an abdomen treatment; it utilized a vacuum immobilization mattress placed under the patient from the head to mid‐thigh constructed at the time of the CT simulation. The planned treatment course was six fractions (10.8 Gy prescribed). The arm position on the ipsilateral side was purposefully imprinted in the vacuum mattress to ensure room for the treatment beams to avoid the arm. At the first fraction it was noted that the vacuum mattress had softened, likely due to an air leak in the mattress causing a loss of vacuum and, hence, rigidity. The vacuum immobilization mattress was determined to have a sufficient remaining imprint for the first treatment and was connected to the facility vacuum during the entirety of the first treatment to maintain rigidity. After the first treatment, the mattress was carefully preserved as best as possible but it was determined a 3D printed immobilization replacement was prudent.

Due to the limits of the build size of the printer, separate 3D printed immobilization structures were printed for the abdomen and the arm; the abdomen structure only extended the length of the treatment volume. Patient support outside of the treatment area was achieved with smaller replacement vacuum mattresses that were formed prior to the second treatment fraction (first fraction using the 3D printed patient immobilization). A similar method of 3D model generation was used as in the lumbar spine case; one difference was that the external contour was expanded 2 mm prior to subtracting from a cubic structure. Another difference was that the shell wall of the abdominal part was increased to 3.6 mm (arm was kept at 2.4 mm) and no scaffolding supports were used. The purpose of increasing the shell wall thickness was to maintain rigidity in the absence of the structural support material. The reason structural supports were not used in this case was an increased confidence in the structural integrity of the PET‐G material and also to minimize the dosimetric effects of the 3D printing material; approximately 50% of the total dose was directed through the 3D printed immobilization material. The effect of the 3D printed immobilization material was considered with respect to skin dose. The original vacuum mattress was about 3 cm thick at the beam entrance (20 × 20 cm^2^, 6 MV). Under those conditions, a skin dose of 65% relative to the dose at d_max_ has been reported.^14^ That percent dose is approximately 2 mm water equivalent thickness using the build‐up dose tables from Olch et al.^15^ For the 3.6 mm 3D printed part, the approximated surface dose is 75%–82%. The total increase in skin dose, however, would be half since the posterior beam delivered half of the total dose. The increase in skin dose was considered an acceptable trade‐off for avoiding treatment delay from replanning.

**FIGURE 3:**
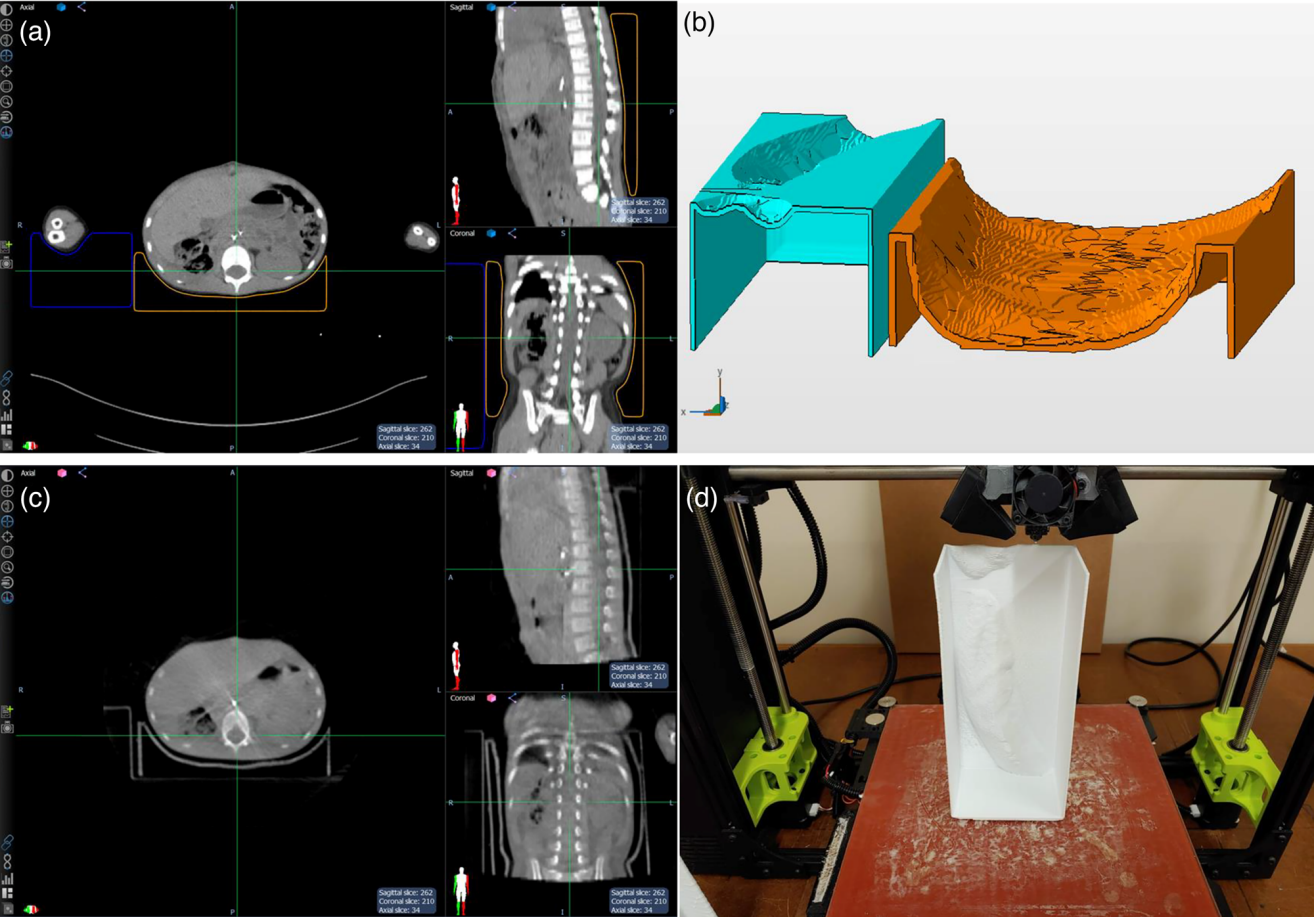
Generation of abdomen and arm immobilization 3D models, (a) axial, sagittal, and coronal view of the cubic structure subtracted from the 2 mm external body contour for the abdomen and arm, (b) 3D models for abdomen (orange) and arm (light blue) immobilization, (c) axial, sagittal, and coronal view of the daily CBCT, and (d) orientation of arm immobilization part on 3D printer.

It can be seen in Figure 3b that three sides of the immobilization 3D models have walls; the reason was to reduce the print time since no scaffolding support material would be required. Figure 3d shows how omitting one wall side eliminated any requirement for overhang scaffolding; the wall opposite of the open side (in contact with the print bed) provides structural support for the lateral walls and helps adhere the plastic to the printer bed during printing.

The daily kV CBCT image guidance couch shifts are reported in the Section 3. Again, daily investigation of the alignment images was performed to ensure the 3D printed devices continued to work as intended. The spine was used to serve as a surrogate for the target location as well as the position of the contralateral kidney, the primary normal tissue avoidance structure. Neither the target volume tissues nor the kidney were readily visible in the CBCT images. The vertebrae in the planning CT were contoured and expanded with 0, 1, 3, 5, 7, 9, and 11 mm expansions. The vertebrae in the daily CBCT images were manually contoured. Similar to the previous case, the image guidance CBCT shift parameters were applied to the CBCT images, and the contours were subtracted from the planning CT vertebrae expansion contours. The volumes were recorded to give a comparison of the interfraction positioning uncertainty margin.

## 3 RESULTS

The accuracy of the patient immobilization is quantified in the following subsections. All statistical uncertainties are reported for one standard deviation. Printing time results are shown in Table 1. Improved print speed is evident for the larger nozzle. For cost comparison purposes, the PET‐G material costs about $20–$60 per kg.

**TABLE 1:** 3D print times and related factors

		Maximal dimensions (cm)	Print time (h)	Nozzle size (mm)	Material mass (g)	Layer thickness (mm)
Headrest	Part 1	20.6 × 14.1 × 6.2	7.0	0.4	250	0.3
	Part 2	20.6 × 13.5 × 6.2	5.3	0.4	190	0.3
Lumbar support		21.9 × 22.6 × 5.2	3.0	1.2	450	0.9
Deflated vacuum bag	Torso	19.5 × 18.7 × 5.6	4.5	1.2	500	0.9
	Arm	22.4 × 10.2 × 7.4	1.8	1.2	190	0.9

### 3.1 Head rest

The clinical CBCT translational registration shifts were 2, 0, and 1 mm in the x (medial‐lateral), y (superior‐inferior), and z (anterior‐posterior) axes, respectively, for the first fraction using the 3D printed head rest; the second fraction had translational shifts of 2, 0, and 2 mm in the x, y, and z axes, respectively. The mean point distance for the skull after image registration was 0.8 ± 1.0 mm and 0.9 ± 0.9 mm for Fractions 1 and 2, respectively. Prior to the use of the 3D printed headrest, no shifts were recorded based on the weekly MV portal images.

### 3.2 Lumbar spine support

The daily MVCT translation registration shifts were analyzed and no statistically significant difference in the registration was observed; the average absolute shifts were 2.1 ± 1.14 mm, 2.9 ± 2.09 mm, and 6.6 ± 6.1 mm in the x, y, and z directions, respectively, prior to the 3D printed lumbar support. With the use of the 3D printed lumbar support the shifts were 1.9 ± 1.7 mm, 1.6 ± 1.2 mm, and 4.2 ± 3.2 mm along the x, y, and z, axes, respectively. During the use of the 3D printed lumbar support, the dose in the lumbar spine region decreased by 3%–4% for the actual treatment delivery. This was determined using an adaptive planning module of the TPS. However, the decrease in dose was not due only to additional attenuation of the 3D printed lumbar support but also due to weight gain (see Section 4).

Comparison of the planned location of the spinal canal to the daily canal position at the level of each vertebral body was performed with the use of the Dice Coefficient. Fractions 1–6 and Fractions 7–18 (before and after the use of 3D printed lumbar support) showed statistically significant improvement in alignment for the spinal canal in the region T12 to L2 with *p*‐values of 0.0018, 00014, and 0.013 for the T12, L1, and L2 regions, respectively. The mean surface point distance also showed statistically significant improvement in the same region with *p*‐values of 0.0042, 0.0029, and 0.026 for the T12, L1, and L2 regions, respectively. No other regions showed statistical significance (*p* = 0.05) in Dice Coefficient or mean surface point distance.

The volume, in cm^3^, of the daily spinal canal contour exceeding the planned spinal canal with incremental margin expansions, in mm, is shown in Figure 5a. Figure 5b shows the daily V95% and the V95% for the composite dose (red and blue lines). The volume of the spinal canal was 151.5 cm^3^.

### 3.3 Deflated vacuum mattress

Daily image CBCT registration shifts ranged from 8 to 16 mm in magnitude for all fractions. No statistical difference was observed between the partially deflated vacuum mattress and the 3D printed replacement. The volume of the vertebrae in the daily CBCT images outside exceeding the planned position with no margin expansion was 23 cm^3^ on the first fraction with the partially deflated vacuum mattress and the average of the subsequent fractions was 20 ± 5 cm^3^. At a 5 mm margin expansion the volume exceeding the margin was 0.9 and 1.1 ± 1.2 cm^3^ for the initial fraction and the average of the subsequent fractions, respectively. No fractions exceeded the 9 mm margin expansion of the planning CT vertebrae contour.

## 4 DISCUSSION

**FIGURE 4:**
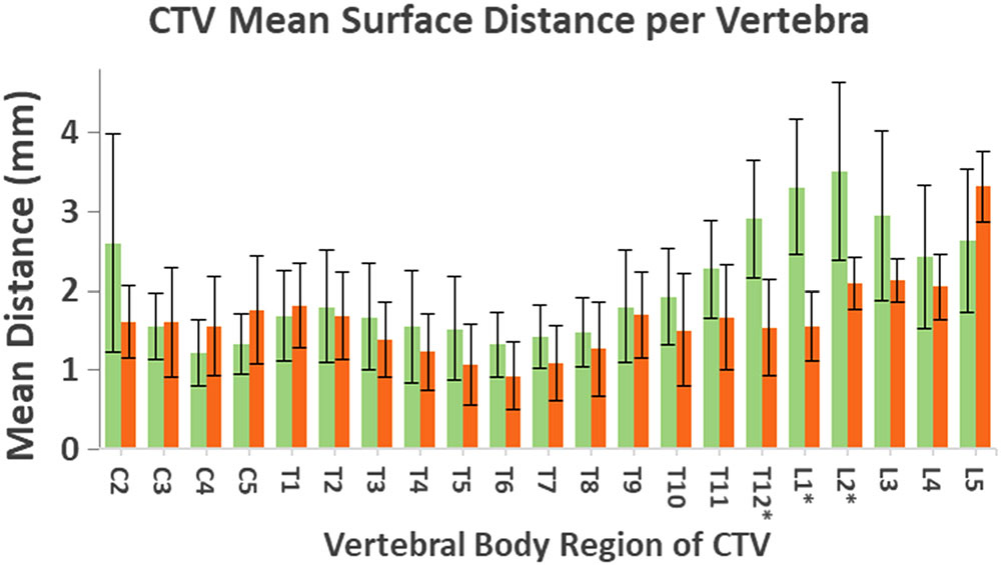
Mean surface distance for entire spine treatment before (green) and after(orange) 3D printed lumbar support. Statistically significant improvement was observed in spinal localization with the 3D printed lumbar support in the regions of the T12, L1, and L2 vertebrae (denoted with asterisk).

The 3D printed immobilization devices resulted in clinically acceptable patient position reproducibility. In Figure 4 it can be seen that the only region where the 3D printed device showed greater positioning uncertainty was in the L5 region. This was considered to be due to patient weight gain during the course of treatment and not related to the 3D printed support. In Figure 2 it can readily be seen that the patient in Panel (b) has larger physical size than in the planning scan in Panel (a); the spatial limits, or zoom, in Panels (a) and (b) are the same. However, the difference in Dice coefficient and mean surface distance remained statistically insignificant. For all cases, the 3D printed immobilization performed as good or better than the traditional methods with statistically significant improvement for the lumbar spine case. Future work will focus on identifying clinical situations where the addition of 3D printed aids can improve patient alignment.

**FIGURE 5:**
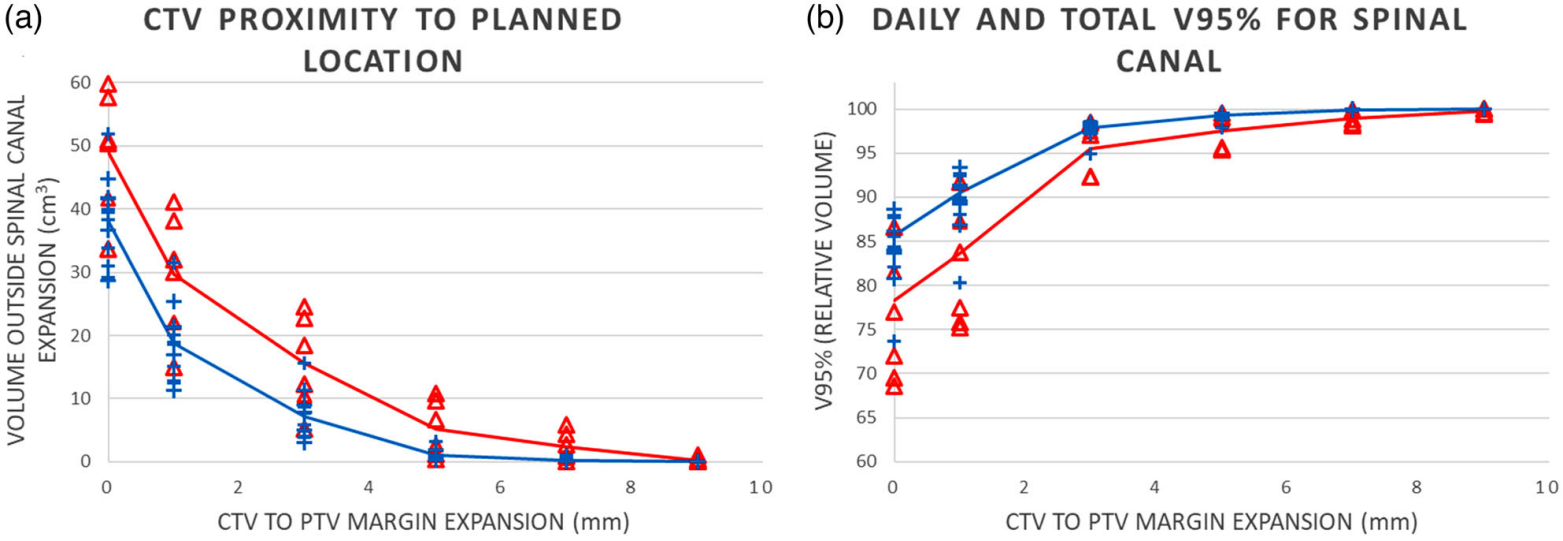
(a) Variation in overlap of planned and daily spinal canal volume and (b) variation in V95% with respect to different margin expansions. Red triangles represent daily results without the 3D printed lumbar support; blue crosses represent daily volumes for fractions where the 3D printed lumbar support was used. The mean volume (a) and the composite dose V95% (b) are shown in blue and red lines indicating with and without the lumbar support, respectively.

Care should be taken when designing the 3D model of an immobilization device using image thresholds and bounding boxes that the patient will actually be able to fit in the device. In Figures 1, 2, 3 the anterior aspect of the bounding boxes was carefully chosen to not result in a device that wraps around the patient anatomy that would prevent the patient from actually being able to be placed in the immobilization device. For cases where the 3D printed device is replacing an existing immobilization device this is more straightforward as similar precautions would have been taken.

The printed head rest case occurred early in the implementation of 3D printing utilization. It was the first attempt to print an object that interlocked with an existing piece of equipment. In Section 2.1 it was mentioned that the pin holes were too narrow and required to be drilled out wider to fit. Based on experience gained since, it is common for the 3D model of existing objects generated from CT images to be smaller in dimension than the actual dimensions. Typically, this is on the order of the resolution of the scan; it is also a function of the diameter of the nozzle used for 3D printing. It is recommended to use as high‐resolution scans as possible. The nozzle size is a tradeoff of printing accuracy and print speed. While it can vary based on a variety of factors including printing material used, printer design, slicing software, and printer settings; generally, the clearance required to make interlocking parts is half the diameter of the printing nozzle. For example, when printing devices that interlock with a 3D printed screw and bolt, prior experience has shown that an offset of half of the nozzle diameter up to the full nozzle diameter is required. In an environment where rapid fabrication is required, it is recommended to perform test prints of the limited volume of the interlocking components in case modifications to the 3D model or print settings are required. In the lumbar spine and the deflated vacuum mattress cases the external contours were expanded by 1 and 2 mm, respectively, based partially from this experience to avoid a situation where the 3D printed object did not fit the patient anatomy.

In the deflated vacuum mattress case, the 3D printed immobilization molds were designed without structural support in order to reduce the amount of plastic the treatment x‐ray beam would pass through and thus reduce the additional x‐ray attenuation in the plastic. Currently, this is the preferred method for patient immobilization; however, it is more technically challenging. For this method the layer thickness, filament plastic flow rate, and wall thickness may need adjustment from printer to printer or even over time with the same printer. In addition, the quality of the plastic can change over time and from manufacturer or even batch to batch.^16^ The dosimetric impact of any 3D printed device should be carefully considered just as any radiotherapy immobilization device. For the headrest case (discussed in Section 2.1) the dosimetric effect of the 3D printed material was not a concern due to the beam arrangement. In retrospect, if the beam arrangement had resulted in the beams passing through the headrest prior to the patient, it would be advised to print with a thin (2–3 mm) shell as in the deflated vacuum bag case to minimize the dosimetric effects. Several studies have been published with dosimetric analysis of several radiotherapy materials and many can be found in recent reviews by Rooney et al.^1^ and Asfia et al.^2^

The use of larger nozzles, and their associated decreased print time, are advantageous when time allowances are compressed. The larger 1.2 mm nozzle was shown to result in faster print times for roughly the same size objects (see Table 1). For comparison, the lumbar support took 3.0 h to print, a standard nozzle size of 0.5 mm would have had an estimated 12.0 h print time using the same slicing software and similar “fast” print settings. The difference in default settings is dependent on the slicing software used but generally default “high resolution,” “normal,” and “fast” modes use layer thicknesses that are ¼, ½, and ¾ of the nozzle diameter. It is recommended when starting the implementation of 3D printing into clinical radiotherapy, to develop high speed printing configurations by choice of nozzle diameter(s), layer thickness, print head speed and acceleration limits. The effect of nozzle size and layer thickness has been discussed in detail for 3D printed radiotherapy bolus.^11^

The reduction of spatial accuracy of the 3D printed object with a larger nozzle (e.g., 1.2 mm) is acceptable for most external beam radiotherapy applications with the possible exception of stereotactic radiotherapy. Since the image resolution of most imaging modalities used in radiation oncology (including those used here) are on the order of millimeters, a difference of 1 mm in 3D printer spatial accuracy is not typically impactful. There are handheld scanners that can be used to generate 3D models with higher resolution but the process of correlating the spatial relationships of the patient anatomy and other treatment devices (headrests, couch top, vacuum bags, etc.) is much more difficult. Furthermore, most clinic staff are experienced in working with CT images. Handheld scanners require experience and technical infrastructure typically outside of radiotherapy clinics and staff.

Infection control of 3D printed objects should also be considered. PET‐G is typically more resistive to degradation than PLA to commercial cleaning solvents. However due to the layered nature of the printing, the surface of the printing is not smooth but rather grooved which can allow for crevices that a standard disinfecting agents may not reach. Therefore, standard linens or plastic cling wrap were placed over the 3D printed object so that the patient's skin did not come in direct contact with the 3D printed plastics. In addition, the material safety data sheets should be consulted for any 3D printing material prior to patient use.

## 5 CONCLUSION

The process of rapid design and 3D printing was described. This can serve as a guide for clinicians in cases when traditional immobilization devices are lost, lose their function, or are deficient or for those starting, or considering starting, implementation of 3D printing in clinical practice. The benefit of the ability of rapid fabrication is that treatment delays can more easily be avoided compared to the alternative of repeat simulation and planning. In light of this, future studies will look at broader conditions where 3D printing can be utilized to improve patient immobilization more frequently than in the types of cases presented here.

## CONFLICT OF INTEREST STATEMENT

None.
